# Protocol to generate human stem cell-derived CD70-directed allogeneic CAR-NKT cells for treating renal cell carcinoma

**DOI:** 10.1016/j.xpro.2025.104340

**Published:** 2026-01-13

**Authors:** Yan-Ruide Li, Lili Yang

**Affiliations:** 1 Department of Microbiology, Immunology & Molecular Genetics, University of California, Los Angeles (UCLA), Los Angeles, CA 90095, USA; 2 Department of Bioengineering, UCLA, Los Angeles, CA 90095, USA; 3 Eli and Edythe Broad Center of Regenerative Medicine and Stem Cell Research, UCLA, Los Angeles, CA 90095, USA; 4 Jonsson Comprehensive Cancer Center, David Geffen School of Medicine, UCLA, Los Angeles, CA 90095, USA; 5 Molecular Biology Institute, UCLA, Los Angeles, CA 90095, USA; 6 Parker Institute for Cancer Immunotherapy, UCLA, Los Angeles, CA 90095, USA; 7 Goodman-Luskin Microbiome Center, UCLA, Los Angeles, CA 90095, USA

**Keywords:** Cell Biology, Cell-based Assays, Cancer, Immunology, Stem Cells, Cell Differentiation, Biotechnology and bioengineering

## Abstract

CD70 has emerged as a promising immunotherapeutic target in renal cell carcinoma (RCC), with both CD70-directed monoclonal antibodies and chimeric antigen receptor (CAR)-based therapies currently under development. Here, we describe a protocol for the generation of human CD70-directed allogeneic CAR-natural killer T (NKT) (^Allo^CAR70-NKT) cells derived from cord blood CD34^+^ hematopoietic stem and progenitor cells (HSPCs) using a clinically guided culture. Furthermore, we describe the therapeutic efficacy of ^Allo^CAR70-NKT cells in mediating cytotoxic activity against RCC cell lines *in vitro*.

For complete details on the use and execution of this protocol, please refer to Li et al.^1^

## Highlights

• Generation of ^Allo^CAR70-NKT cells from hematopoietic stem and progenitor cells
• *Ex vivo* differentiation of ^Allo^CAR70-NKT cells via a clinically guided culture method
• Evaluation of antitumor capacity of ^Allo^CAR70-NKT cells against renal cell carcinoma

## Abstract (editor highlights)

Publisher’s note: Undertaking any experimental protocol requires adherence to local institutional guidelines for laboratory safety and ethics.

## Graphical abstract

**Figure:**
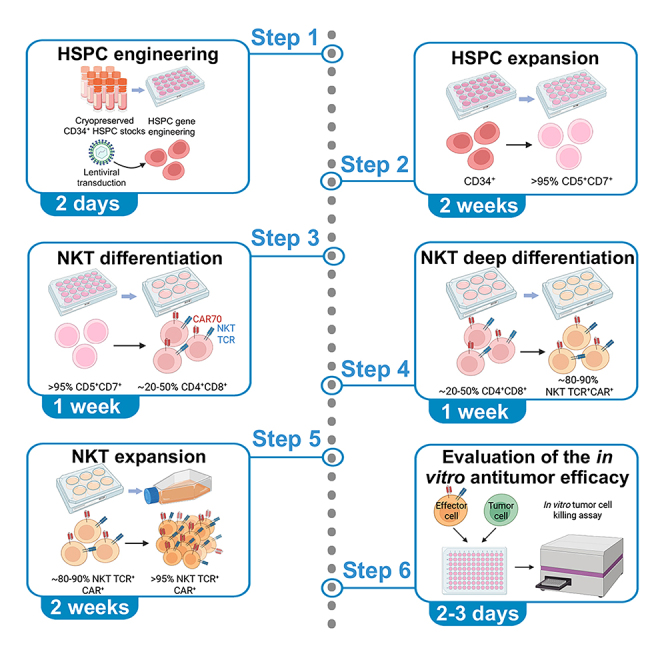

## Teaser

CD70 has emerged as a promising immunotherapeutic target in renal cell carcinoma (RCC), with both CD70-directed monoclonal antibodies and chimeric antigen receptor (CAR)-based therapies currently under development. Here, we describe a protocol for the generation of human CD70-directed allogeneic CAR-natural killer T (NKT) (^Allo^CAR70-NKT) cells derived from cord blood CD34^+^ hematopoietic stem and progenitor cells (HSPCs) using a clinically guided culture. Furthermore, we describe the evaluation of the therapeutic efficacy of ^Allo^CAR70-NKT cells in mediating cytotoxic activity against RCC cell lines *in vitro*.

## Before you begin

This protocol outlines a comprehensive workflow for the generation of human ^Allo^CAR70-NKT cells from cord blood–derived HSPCs for the treatment of RCC. The generated ^Allo^CAR70-NKT cells are also capable of recognizing and targeting other CD70-expressing malignancies, such as glioblastoma, non-Hodgkin lymphoma, and acute myeloid leukemia.^2^^,^^3^ Furthermore, the protocol details procedures for evaluating antitumor efficacy using *in vitro* tumor cell killing assays with RCC cell lines that exhibit varying levels of CD70 expression, thereby modeling different antigen-density scenarios. Overall, this protocol provides a clinically translatable framework for developing off-the-shelf CAR-NKT cell-based immunotherapies against RCC and other CD70^+^ tumors.

### Innovation

This protocol describes an innovative method for generating allogeneic CAR-NKT cells from human HSPCs, offering a scalable, off-the-shelf platform for adoptive cell therapy. Unlike conventional approaches that rely on peripheral blood mononuclear cells (PBMCs) from healthy donors or cancer patients—where NKT cells represent only a minute fraction of total lymphocytes—this system enables robust *ex vivo* differentiation of NKT cells from cord blood–derived HSPCs, producing cell products with high yield, purity, and reproducibility.^4^

The protocol is highly modular, allowing for additional genetic modifications at the HSPC stage to introduce desirable traits before NKT cell differentiation. Examples include cytokine engineering to enhance persistence and effector function, and targeted checkpoint-gene disruption to mitigate exhaustion and improve antitumor potency.^5^^,^^6^ This flexibility provides a foundation for developing next-generation CAR-NKT platforms with tunable immune behavior.

Compared with conventional donor-derived methods, which are limited by donor variability, low NKT cell frequency, and manufacturing constraints, the described workflow achieves a clinically relevant scale of CAR-NKT production suitable for broader patient access.^7^^,^^8^^,^^9^ The resulting cells maintain potent cytotoxicity, uniform phenotype, and compatibility for allogeneic use, thereby overcoming key translational bottlenecks in CAR-based immunotherapy. Overall, this approach represents a significant advancement in the generation of universal, off-the-shelf CAR-NKT cells with customizable genetic features and strong potential for clinical application.

### Institutional permissions

Purified cord-blood-derived human CD34^+^ HSPCs were commercially obtained from the HemaCare. Healthy donor peripheral blood mononuclear cells (PBMCs) were obtained from the UCLA/CFAR Virology Core Laboratory without identification information under federal and state regulations.

### Human RCC cell line engineering

**Timing: 3 days**

***Note:*** This protocol outlines the development of a series of human RCC tumor cell lines designed for use in *in vitro* tumor cell killing assays to assess antitumor efficacy of ^Allo^CAR70-NKT cells. It can be adapted for a wide range of tumor cell lines beyond human RCC cell lines.

1. Culture human RCC cell lines (e.g., 786-O and ACHN) in R10 medium.
2. Seed 0.5–1 x 10^6^ tumor cells into one well of a 6-well plate.
3. Thaw and add the concentrated Lenti/FG virus supernatant.
  ***Note:*** FG denotes firefly luciferase and enhanced green fluorescent protein dual-reporters. FG-engineered RCC cells enable monitoring by flow cytometry as well as by quantitative luminescence measurements.^10^
  a. Gently mix the thawed supernatant by pipetting (do not vortex), then dispense it directly into the wells.
  b. Gently rock the plate to ensure mixing.
  c. After 16–24 h of incubation, add another 1 mL of fresh R10 medium to avoid tumor cell death.
4. Culture the RCC tumor cells in a humidified CO_2_ incubator at 37°C for 3 days. Regularly split the tumor cells to prevent overgrowth.

***Note:*** Splitting methods differ among RCC cell lines; therefore, use the cell line–specific procedure recommended by the supplier (ATCC).

5. Assess FG expression using either flow cytometry or fluorescence microscopy.
6. Isolate GFP^+^ tumor cells by FACS-sorting to achieve a 100% FG-labeled population. These enriched cells are suitable for subsequent *in vitro* and *in vivo* experiments.

***Note:*** Over extended periods of culture, tumor cells may gradually lose FG expression, which can compromise the accuracy and reliability of experimental results. For this reason, it is important to routinely assess GFP levels. If a decline in GFP expression is detected, the cells should either be re-sorted to enrich for GFP^+^ cells or re-engineered to restore stable FG expression.

## Key resources table

**Table:** 

REAGENT or RESOURCE	SOURCE	IDENTIFIER
**Antibodies**		

Anti-human TCR αβ (Clone IP26); 1:50 dilution	BioLegend	CAT#306716, RRID: AB_1953257
Anti-human CD3 (clone HIT3a); 1:500 dilution	BioLegend	CAT#300329, RRID: AB_10552893
Anti-human CD4 (Clone OKT4); 1:400 dilution	BioLegend	CAT#317414, RRID: AB_571959
Anti-human CD5 (Clone L17F12); 1:500 dilution	BioLegend	CAT#364014, RRID: AB_2565284
Anti-human CD7 (Clone 4H9/CD7); 1:500 dilution	BioLegend	CAT#395606, RRID: AB_2888714
Anti-human CD8 (Clone SK1); 1:300 dilution	BioLegend	CAT#344714, RRID: AB_2044006
Anti-human CD34 (Clone 581); 1:500 dilution	BioLegend	CAT#343520, RRID: AB_1937269
Anti-human CD45 (Clone HI30); 1:500 dilution	BioLegend	CAT#982318, RRID: AB_2888786
Anti-human CD70 (Clone WM53); 1:50 dilution	BioLegend	CAT#355109, RRID: AB_2562480
Mouse Fc Block (anti-mouse CD16/32); 1:50 dilution	BD Biosciences	CAT#553142, RRID: AB_394657
Human Fc Receptor Blocking Solution (TruStain FcX); 1:50 dilution	BioLegend	CAT#422302, RRID: AB_2818986
Anti-human IFN-γ (ELISA, capture; Clone NIB42)	BD Biosciences	CAT#551221, RRID: AB_394099
Anti-human IFN-γ (ELISA, detection; Clone 4S.B3)	BD Biosciences	CAT#554550, RRID: AB_395472
Anti-human TNF-α (ELISA, capture; Clone MAb1)	BD Biosciences	CAT#551220, RRID: AB_394098
Anti-human TNF-α (ELISA, detection; Clone MAb11)	BD Biosciences	CAT#554511, RRID: AB_395442
Anti-human IL-2 (ELISA, capture; Clone MQ1-17H12)	BD Biosciences	CAT#554563; RRID: AB_398570
Anti-human IL-2 (ELISA, detection; Clone B33-2)	BD Biosciences	CAT#555040; RRID: AB_395666
Anti-human IL-4 (ELISA, capture; Clone 8D4-8)	BD Biosciences	CAT#554515; RRID: AB_398567
Anti-human IL-4 (ELISA, detection; Clone MP4-25D2)	BD Biosciences	CAT#554483; RRID: AB_395422
Anti-human IL-15 (ELISA, capture; Clone G243-935)	BD Biosciences	CAT#554712; RRID: AB_2561319
Anti-human IL-15 (ELISA, detection; Clone G243-886)	BD Biosciences	CAT#554713; RRID: AB_2561320
Anti-human TCR Vα24-Jβ18 (Clone 6B11); 1:50 dilution	BD Biosciences	CAT#552825, RRID: AB_394478
Anti-human iNKT TCR Vβ11 (Clone C2); 1:20 dilution	Beckman Coulter	Product#A66905

**Bacterial and virus strains**		

Lenti/iNKT-CAR70-IL-15	This paper	N/A
Lenti/FG	This paper	N/A

**Biological samples**		

Human peripheral blood mononuclear cells (PBMCs)	UCLA	N/A
Cord blood CD34^+^ stem/progenitor cells	HemaCare	N/A

**Chemicals, peptides, and recombinant proteins**		

Streptavidin-HRP conjugate	Invitrogen	CAT#SA10001
Human IFN-γ (ELISA, standard)	eBioscience	CAT#29-8319-65
Human TNF-α (ELISA, standard)	eBioscience	CAT#29-8329-65
Human IL-2 (ELISA, standard)	eBioscience	CAT#29-8029-65
Human IL-4 (ELISA, standard)	eBioscience	CAT#39-8049-65
Tetramethylbenzidine (TMB)	KPL	CAT#5120-0053
α-Galactosylceramide (KRN7000)	Avanti Polar Lipids	SKU#867000P-1mg
Recombinant human IL-2	Peprotech	CAT#200-02
Recombinant human IL-3	Peprotech	CAT#200-03
Recombinant human IL-4	Peprotech	CAT#200-04
Recombinant human IL-7	Peprotech	CAT#200-07
Recombinant human IL-13	Peprotech	CAT#200-13
Recombinant human IL-15	Peprotech	CAT#200-15
Recombinant human IL-21	Peprotech	CAT#200-21
Recombinant human IFN-γ	Peprotech	CAT#300-02
Recombinant human Flt3-Ligand	Peprotech	CAT#300-19
Recombinant human SCF	Peprotech	CAT#300-07
Recombinant human TPO	Peprotech	CAT#300-18
Recombinant human GM-CSF	Peprotech	CAT#300-03
Recombinant human M-CSF	Peprotech	CAT#300-25
L-ascorbic acid 2-phosphate	Sigma	CAT#A8960-5G
B27™ Supplement (50X), serum free	ThermoFisher	CAT#17504044
RPMI1640 cell culture medium	Corning Cellgro	CAT#10-040-CV
DMEM cell culture medium	Corning Cellgro	CAT#10-013-CV
Fetal Bovine Serum (FBS)	Sigma	CAT#F2442
MACS BSA stock solution	Miltenyi	CAT#130-091-376
30% BSA	Gemini	CAT#700-110-100
Penicillin-Streptomycine-Glutamine (P/S/G)	Gibco	CAT#10378016
Penicillin: streptomycin (pen:strep) solution (P/S)	Gemini Bio-products	CAT#400-109-100
MEM non-essential amino acids (NEAA)	Gibco	CAT#11140050
HEPES Buffer Solution	Gibco	CAT#15630080
Sodium Pyruvate	Gibco	CAT#11360070
Beta-Mercaptoethanol	Sigma	SKU#M6250
Normocin	Invivogen	CAT#ant-nr-2
Fixable Viability Dye eFluor506	eBioscience	CAT#65-0866-14
Cell Fixation/Permeabilization Kit	BD Biosciences	CAT#554714
RetroNectin recombination human fibronectin fragment, 2.5mg	Takara	CAT#T100B
10% neutral-buffered formalin	Richard-Allan Scientific	CAT#5705
D-Luciferin Potassium Salt Bioluminescent Substrate	Revivi	CAT#122799-100MG
Phosphate Buffered Saline (PBS) pH 7.4 (1X)	Gibco	CAT#10010-023
Formaldehyde	Sigma-Aldrich	CAT#F8775
Phorbol-12-myristate-13-acetate (PMA)	Sigma	CAT#524400
Ionomycin, Calcium salt, *Streptomyces conglobatus*	Sigma	CAT#407952
ImmunoCult™ Human CD3/CD28/CD2 T Cell Activator	Stem Cell Technologies	CAT#10970
MethoCult™H4330 MethycelluloseBased Medium	Stem Cell Technologies	CAT#04330
CTS™ OpTmizer™ T-Cell Expansion SFM (no phenol red, bottle format, cat.	Thermo Fisher Scientific	CAT#A3705001
CryoStor ® Cell Cryopreservation Media CS10	MilliporeSigma	CAT#C2874

**Critical commercial assays**		

Human CD34 MicroBeads Kit	Miltenyi Biotec	CAT#130-046-703
Human Anti-NKT MicroBeads	Miltenyi Biotec	CAT#130-094-842
Dynabeads Human T-Activator CD3/CD28	ThermoFisher	CAT#11131D
Cryostor cell cryopreservation media	Sigma	CAT#C2874-100ML
StemSpan”” Lymphoid Differentiation Coating Material (100X)	Stem Cell Technologies	CAT#9925
StemSpan” SFEM II	Stem Cell Technologies	CAT#9605
ImmunoCult” Human CD3/CD28/CD2 T Cell Activator	Stem Cell Technologies	CAT#10970
TransIT-Lenti Transfection Reagent	Mirus Bio	CAT#MIR 6600
Amicon Ultra-15 Centrifugal Filter Unit	Sigma	CAT#UFC910024
Human IL-17A ELISA MAX Deluxe Kit	BioLegend	CAT#433915

**Experimental models: Cell lines**		

Human renal cell carcinoma cell line 786-O	ATCC	CRL-1932
Human renal cell carcinoma cell line ACHN	ATCC	CRL-1611
Human chronic myelogenous leukemia cell line K562	ATCC	CRL-2974
Human artificial APC cell line	This paper	N/A
Human renal cell carcinoma cell line 786-O-FG	This paper	N/A
Human renal cell carcinoma cell line ACHN-FG	This paper	N/A

**Recombinant DNA**		

Vector: parental lentivector pMNDW	Giannoni et al.^11^ Zhu et al.^12^	N/A

**Software and algorithms**		

FlowJo Software	FlowJo	https://www.flowjo.com/solutions/flowjo/downloads
Prism 9	Graphpad	https://www.graphpad.com/scientific-software/prism/

## Materials and equipment

### C10 medium

To formulate C10 medium for NKT-cell culture, combine the following reagents: 100 mL fetal bovine serum (FBS), 10 mL Penicillin-Streptomycin-Glutamine (P/S/G, 100×), 10 mL MEM non-essential amino acids (100×), 10 mL HEPES (1 M), 10 mL sodium pyruvate (100 mM), 10 mL 5 mM β-mercaptoethanol, and 2 mL Normocin (500×). Add these components to 848 mL of RPMI to obtain a total volume of 1 L. Mix thoroughly in a sterile 1-L bottle, then sterilize the medium by passing it through a 0.22-μm filter into the same container.

**Table:** 

Reagent	Final concentration	Amount
RPMI	N/A	848 mL
FBS	10%	100 mL
P/S/G (100x)	1x	10 mL
MEM NEAA (100x)	1x	10 mL
HEPES Buffer Solution (1 M)	10 mM	10 mL
Sodium Pyruvate (100 mM)	1 mM	10 mL
β-ME (5 mM)	50 μM	10 mL
Normocin (500x)	1x	2 mL
**Total**	**N/A**	**1,000 mL**

Store the prepared C10 medium at 4°C in a dedicated tissue-culture refrigerator; it remains suitable for use for up to one month.

### R10 medium

To prepare R10 medium for culturing human RCC tumor cells, thaw 100 mL FBS, 10 mL P/S/G (100×), and 2 mL Normocin (500×) in a 37°C water bath. Separately, measure 888 mL of RPMI and add it to the thawed reagents to obtain a final volume of 1 L. See the table below for specific reagent amounts. Transfer the mixture into a sterile, autoclaved 1-L bottle and sterilize it by filtering through a 0.22-μm filter top.

**Table:** 

Reagent	Final concentration	Amount
RPMI	N/A	888 mL
FBS	10%	100 mL
P/S/G (100x)	1x	10 mL
Normocin (500x)	1x	2 mL
**Total**	**N/A**	**1,000 mL**

Store the completed R10 medium at 4°C in the tissue-culture refrigerator; it remains usable for up to one month.

### HSPC culture medium

Prepare the HSPC culture medium by supplementing X-VIVO™ 15 serum-free hematopoietic stem cell medium with 50 ng/mL stem cell factor (SCF), 50 ng/mL Flt3 ligand, 50 ng/mL thrombopoietin (TPO), and 20 ng/mL interleukin-3 (IL-3). Attach a 0.22-μm filter unit to a sterile bottle and sterilize the prepared medium by filtration to ensure aseptic conditions. For cell seeding, resuspend 1 × 10^4^ thawed human CD34^+^ HSPCs in 300 μL of the prepared medium.

**Table:** 

Reagent	Final concentration	Amount
X-VIVO™ 15 serum-free hematopoietic stem cell medium	N/A	50 mL
Stem cell factor (SCF)	50 ng/mL	2,500 ng
Flt3 ligand	50 ng/mL	2,500 ng
Thrombopoietin (TPO)	50 ng/mL	2,500 ng
IL-3	20 ng/mL	1,000 ng
**Total**	**N/A**	**50 mL**

The freshly prepared medium can be stored at 4°C for up to two weeks without loss of performance. To preserve experimental consistency and cell viability, discard any medium stored beyond this period.

### HSPC expansion medium

After transduction of human CD34^+^ HSPCs, prepare the HSPC expansion medium for subsequent culture. To formulate the expansion medium, supplement StemSpan™ SFEM II Medium with 10× StemSpan™ Lymphoid Progenitor Expansion Supplement, following the stock concentrations and volumes indicated in the accompanying table. Attach a 0.22-μm filter unit to a sterile bottle and sterilize the prepared medium by filtration to ensure aseptic conditions. For culture setup, resuspend 2 × 10^4^ transduced CD34^+^ HSPCs per 500 μL of the prepared HSPC expansion medium.

**Table:** 

Reagent	Final concentration	Amount
StemSpan SFEM II serum-free medium	N/A	45 mL
StemSpan Lymphoid Progenitor Expansion Supplement (10×)	1x	5 mL
**Total**	**N/A**	**50 mL**

The freshly prepared medium can be stored at 4°C for up to two weeks without loss of quality. To maintain optimal experimental reproducibility and cell viability, discard any medium that has been stored for longer than this period.

### NKT differentiation medium

Prepare the NKT cell differentiation medium by supplementing StemSpan™ SFEM II Medium with 10× StemSpan™ Lymphoid Progenitor Maturation Supplement, following the concentrations and volumes outlined in the accompanying table. Attach a 0.22-μm filter unit to a sterile bottle and sterilize the prepared medium by filtration to ensure aseptic conditions. For culture setup, resuspend 1 × 10^5^ Stage 2 NKT cells per 500 μL of the prepared differentiation medium.

**Table:** 

Reagent	Final concentration	Amount
StemSpan™ SFEM II Medium	N/A	45 mL
StemSpan™ Lymphoid Progenitor Maturation Supplement (10x)	1x	5 mL
**Total**	**N/A**	**50 mL**

The freshly prepared medium can be stored at 4°C for up to two weeks while retaining optimal performance. To ensure experimental reproducibility and medium integrity, discard any medium stored beyond this two-week period.

### NKT deep differentiation medium

Formulate the deep differentiation medium for NKT cells by enriching StemSpan™ SFEM II Medium with 10× StemSpan™ Lymphoid Progenitor Maturation Supplement and 10 ng/mL recombinant human IL-15. At the initiation of the culture, add ImmunoCult™ Human CD3/CD28/CD2 T Cell Activator at 12.5 μL/mL to drive deep NKT differentiation. Refer to the accompanying table for stock concentrations and the corresponding reagent volumes. Sterilize the complete medium using a 0.22-μm filter unit attached to a sterile bottle to maintain aseptic conditions. For culture initiation, resuspend 5 × 10^5^ to 1 × 10^6^ Stage 2 NKT cells per milliliter of the prepared medium.

**Table:** 

Reagent	Final concentration	Amount
StemSpan™ SFEM II Medium	N/A	45 mL
StemSpan™ Lymphoid Progenitor Maturation Supplement (10x)	1x	5 mL
Human IL-15	10 ng/mL	500 ng
**Total**	**N/A**	**50 mL**

The medium may be stored at 4°C for up to two weeks with retained functionality; discard any remaining medium afterward to ensure consistency and reproducibility across experiments.

### NKT cell expansion medium

Prepare the NKT cell expansion medium for culturing highly differentiated ^Allo^CAR70-NKT cells by supplementing homemade C10 medium with 10 ng/mL human IL-7 and 10 ng/mL human IL-15. Sterilize the complete medium by passing it through a 0.22-μm filter attached to a sterile bottle to maintain aseptic conditions. Adjust the final cell resuspension density based on the specific expansion protocol being followed; detailed instructions are provided in the corresponding procedure section.

**Table:** 

Reagent	Final concentration	Amount
C10 medium	N/A	50 mL
Human IL-7	10 ng/mL	500 ng
Human IL-15	10 ng/mL	500 ng
**Total**	**N/A**	**50 mL**

Freshly prepared medium may be stored at 4°C for up to two weeks without loss of performance. Discard any remaining medium after this period to ensure consistency and experimental reliability.

## Step-by-step method details

### Generation of ^Allo^CAR70-NKT cells: HSPC engineering

**Timing: 2 days**

The *ex vivo* generation of ^Allo^CAR70-NKT cells from cord blood–derived CD34^+^ HSPCs comprises five sequential stages: (1) HSPC engineering, (2) HSPC expansion, (3) NKT differentiation, (4) NKT deep differentiation, and (5) NKT expansion (Table 1).^13^^,^^14^ This section details the first stage—HSPC engineering, which involves the genetic modification of HSPCs to introduce the CD70-directed CAR construct and associated molecular components necessary for downstream NKT lineage differentiation.

1. Thaw Retronectin (RN) stock (1 μg/μL) stored at −20°C.
2. Dilute RN to a final concentration of 20 μg/mL in sterile 1× PBS. Dispense 1 mL of the diluted solution into each well of a 6-well, non–tissue-culture–treated plate.
3. Incubate the plate at 20°C–25°C for 2 hours to allow RN coating.
4. Aspirate the RN solution and replace it with 1 mL of 2% (wt/vol) BSA in PBS to block nonspecific binding. Incubate for 30 minutes at 20°C–25°C.
5. Remove the blocking solution by aspiration and wash each well with 2 mL of 1× PBS. Use the RN-coated plate the same day.

***Note:*** RN-coated plate can be used up to a week if stored at 4 degrees and properly sealed.

6. Thaw human CD34^+^ HSPCs rapidly in a 37°C water bath. Troubleshooting 1.

**CRITICAL:** During HSPC culture, no antibiotics are included in the culture medium; therefore, it is essential to maintain strict aseptic conditions within the incubator to minimize the risk of microbial contamination. Monitor the cultures daily to assess cell morphology, viability, and density, and to confirm the absence of contamination throughout the culture period.

7. Add 500 μL of pre-warmed X-VIVO 15 medium dropwise to the thawed CD34^+^ HSPCs to reduce osmotic stress. Transfer these HSPCs to a 15 mL conical tube.
8. Centrifuge at 300 × g for 10 minutes and aspirate the supernatant.
9. Resuspend the pellet in 10 mL of HSPC culture medium and determine viable cell count.
10. Centrifuge again at 300 × g for 10 minutes, remove the supernatant, and resuspend cells at 1 × 10^6^ cells/mL in Stage 0 medium.
11. Remove PBS from the RN-coated wells and seed 1 mL of cell suspension per well.
12. Incubate the plate at 37°C with 5% CO_2_ for 12–18 hours to allow the cells to adhere.
13. Thaw concentrated viral supernatant (Lenti/iNKT-CAR70-IL-15^1^) sufficient to achieve an MOI = 100 or a final concentration of 1 × 10^8^ IFU/mL. Troubleshooting 2.

***Note:*** We chose an MOI = 100 based on prior internal experience using similar concentrated lentiviral preps and CD34^+^ HSPCs in our laboratory, where this input reliably produced robust transgene marking with acceptable cell viability for downstream functional assays.^14^

14. Gently mix the thawed viral supernatant by pipetting (do not vortex) and add it directly to the wells. Tilt or rock the plate gently to distribute the virus evenly.
15. Incubate the plate at 37°C, 5% CO_2_ for 24 hours.
16. After 24 hours, gently pipette to detach cells and transfer them to a 15-mL conical tube.
17. Rinse each well with an equal volume of cold HSPC culture medium to recover any remaining cells, pooling all washes into the same tube.
18. Examine under a microscope to ensure complete cell collection. Perform additional cold washes if necessary.
19. Centrifuge at 300 × g for 10 minutes and aspirate the medium.
20. Resuspend the pellet in 10 mL of HSPC culture medium and count viable cells.

***Note:*** In order to assess transduction efficiency on HSPCs, 1 × 10^5^ HSPCs can be cultured in 1 mL of Stage 0 medium for another 3 days and then analyzed by flow cytometry. Detect intracellular human iNKT TCR Vβ11, identifying Vβ11^+^ cells as successfully transduced.

***Note:*** Because HSPCs do not express surface CD3, the iNKT TCR complex is not present on the cell surface and thus cannot be detected by surface staining. Consequently, iNKT TCR can only be identified through intracellular staining.

21. Centrifuge the cells at 300 × g for 10 minutes, remove the supernatant, and resuspend in HSPC expansion medium at 2 × 10^4^ cells per 500 μL for subsequent differentiation and culture.

**Table 1:** Detailed workflow and culture conditions for ^Allo^CAR70-NKT cell differentiation

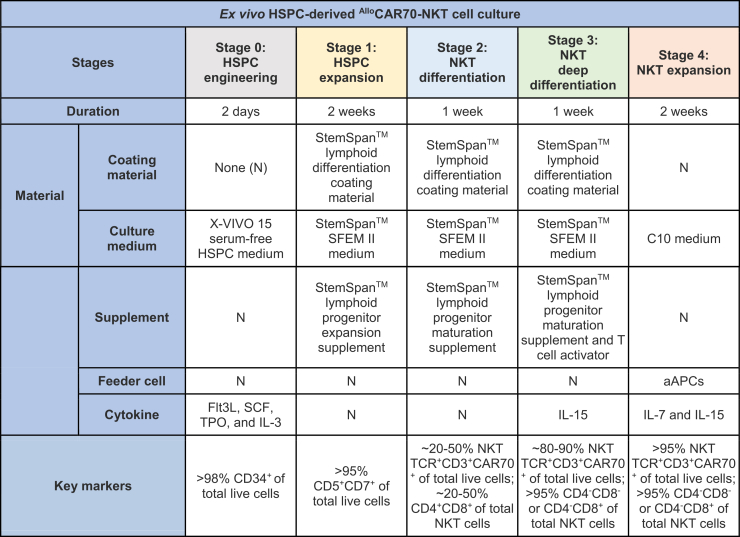

### Generation of ^Allo^CAR70-NKT cells: HSPC expansion

**Timing: 2 weeks**

This section details the second stage of the *ex vivo* generation of ^Allo^CAR70-NKT cells—HSPC expansion.

22. Thaw StemSpan™ Lymphoid Differentiation Coating Material at 20°C–25°C and add 500 μL of the material to each well of a CELLSTAR® 24-well non–tissue culture–treated plate.
23. Incubate the non–tissue culture–treated plate for 2 hours at 20°C–25°C or 16–20 hours at 4°C.

***Note:*** If the coating material is adhesive, make sure to pipette several times to achieve complete coverage of the well.

24. Remove the coating solution and rinse each well gently with 1 mL of 1x PBS to eliminate residual coating material.
25. Transfer engineered HSPCs into the coated wells at a density of 500 μL per well and culture for 3 days at 37°C, 5% CO_2_.
26. On day 4, supplement each well with an additional 500 μL of HSPC expansion medium and continue culturing for 4 days.

***Note:*** At this stage, HSPCs generally maintain a low proliferation rate and do not typically overgrow. However, if overgrowth is observed—indicated by medium color change (yellowing) or cell confluence exceeding 70% under microscopic examination—collect the cells, centrifuge at 300 × g for 10 min, and resuspend them in fresh HSPC expansion medium. Re-seed the cells evenly into two wells to restore optimal culture density.

27. On day 7, remove half of the existing medium and replace it with 500 μL of fresh HSPC expansion medium. Continue culturing the cells for an additional 4 days.

**CRITICAL:** A half-medium change helps maintain a stable and supportive culture environment for HSPCs. By removing only half of the old medium and replacing it with fresh expansion medium, nutrients and growth factors are replenished while beneficial cytokines and cell-derived signals already present in the culture are preserved. This method prevents sudden shifts in concentration, reduces cellular stress, and supports sustained proliferation and viability over extended culture periods.

28. On day 11, repeat the half medium change and culture for 3 more days.
29. On day 14, collect cells by gentle pipetting, determine viable cell counts, and centrifuge at 300 × g for 10 minutes.

***Note:*** The growth rate of NKT cells can vary depending on the cord blood HSPC batch and the efficiency of lentiviral transduction. Therefore, daily monitoring is essential to prevent overgrowth and to ensure the culture remains healthy.

30. Resuspend the cell pellet in NKT differentiation medium at a concentration of 1 × 10^5^ cells/mL for use in the next stage.
31. If total ^Allo^CAR70-NKT cell number exceeds culture capacity, process 1–2 × 10^5^ cells for differentiation and cryopreserve remaining cells at 2 × 10^5^ cells in 500 μL freezing medium for later use.

***Note:*** During the *ex vivo* generation of ^Allo^CAR70-NKT cells, samples can be collected weekly for flow cytometric analysis to assess phenotype and differentiation. The cells are characterized by the expression profile CD45^+^CD3^+^TCRαβ^+^ NKT TCR (6B11)^+^CAR70^+^. CAR70 expression was detected using an anti-CD27 antibody, as the CAR70 construct was designed using CD27 as the ligand-binding domain to specifically recognize CD70-expressing target cells.^1^

### Generation of ^Allo^CAR70-NKT cells: NKT differentiation

**Timing: 1 week**

This section details the third stage of the *ex vivo* generation of ^Allo^CAR70-NKT cells—NKT differentiation.

32. Thaw StemSpan™ Lymphoid Differentiation Coating Material at 20°C–25°C and add 1 mL of the coating material to each well of a Falcon non–tissue culture–treated 6-well polystyrene plate.
33. Incubate the non–tissue culture–treated plate for 2 hours at 20°C–25°C or 16–20 hours at 4°C.

***Note:*** If the coating material is adhesive, make sure to pipette several times to achieve complete coverage of the well.

34. Remove the coating solution and rinse each well with 1 mL of 1x PBS to eliminate any residual coating material.
35. Transfer ^Allo^CAR70-NKT cells into the coated wells at 2 mL per well using NKT cell differentiation medium.
36. Culture the cells for 1 week at 37°C, 5% CO_2_ to initiate ^Allo^CAR70-NKT differentiation.
37. During this 1-week period, passage the cells two to three times to maintain an optimal density of 0.5–1 x 10^6^ cells/mL.
38. For each passage, gently pipette the cells up and down five times to detach and resuspend them.
  a. Centrifuge at 300 x g for 10 minutes.
  b. Aspirate the supernatant.
  c. Resuspend the pellet in fresh NKT differentiation medium at a concentration of 1 x 10^5^ cells/mL.
39. At the end of this stage, collect approximately 1 × 10^5^ cells for flow cytometric analysis to assess NKT differentiation.

***Note:*** Stain ^Allo^CAR70-NKT cells with NKT TCR 6B11, CD3, TCRαβ, CAR70, CD4, CD8, and viability dye (eFluor 506; e506). The expected phenotype of differentiated NKT cells is: >70% CAR-NKT cells (CD3^+^TCRαβ^+^6B11^+^CAR70^+^), 20%–50% CD4^+^CD8^+^ double-positive cells, and >90% viability (e506^-^ population).

### Generation of ^Allo^CAR70-NKT cells: NKT deep differentiation

**Timing: 1 week**

This section details the fourth stage of the *ex vivo* generation of ^Allo^CAR70-NKT cells—NKT deep differentiation.

40. Thaw StemSpan™ Lymphoid Differentiation Coating Material at 20°C–25°C and add 1 mL of the coating material to each well of a Falcon® non–tissue culture-treated 6-well polystyrene plate.
41. Incubate the non–tissue culture-treated plate for 2 hours at 20°C–25°C or 16–20 hours at 4°C.

***Note:*** If the coating material is adhesive, make sure to pipette several times to achieve complete coverage of the well.

42. Remove the coating solution and rinse each well with 1 mL of 1x PBS to eliminate any residual coating material.
43. One week after initiating NKT differentiation, collect ^Allo^CAR70-NKT cells from the previous stage.
44. Determine viable cell counts, centrifuge at 300 × g for 10 minutes, and resuspend cells in NKT deep differentiation medium at a concentration of 5 × 10^5^ cells/mL.
45. Supplement the medium with ImmunoCult™ Human CD3/CD28/CD2 T Cell Activator to achieve a final concentration of 12.5 μL/mL.
46. Seed 2 mL of cell suspension per well into the coated 6-well plates and incubate at 37°C, 5% CO_2_ for 1 week. During this 1-week culture period, passage cells two to three times to maintain an optimal density of 0.5–1 × 10^6^ cells/mL.
47. For each passage, gently pipette the culture up and down five times to detach and resuspend the cells.
  a. Centrifuge at 300 × g for 10 minutes.
  b. Discard the supernatant.
  c. Resuspend cells in fresh NKT deep differentiation medium at a concentration of 5 × 10^5^ cells/mL.
48. At the end of this stage, collect approximately 1 × 10^5^ cells for flow cytometric analysis to assess deep differentiation.

***Note:*** Stain cells with the following markers: NKT TCR 6B11, CD3, TCRαβ, CAR70, CD4, CD8, and viability dye (e506). The expected phenotype of differentiated ^Allo^CAR70-NKT cells is: >95% NKT cells (CD3^+^TCRαβ^+^6B11^+^CAR70^+^), cells primarily progress to CD4^-^CD8^-^ or CD4^-^CD8^+^ stages, and >90% viability (e506^-^ cell population).

### Generation of ^Allo^CAR70-NKT cells: NKT expansion

**Timing: 2 weeks**

This section details the fifth stage of the *ex vivo* generation of ^Allo^CAR70-NKT cells—NKT expansion.

49. Before initiating NKT cell expansion, collect NKT cells from the previous stage and determine the total viable cell count.
50. Calculate the required number of artificial antigen-presenting cells (aAPCs) based on an NKT:aAPC ratio of 1:1–1:2.

***Note:*** The aAPC was created by genetically modifying the K562 human chronic myelogenous leukemia cell line (ATCC, CCL-243) to overexpress human CD80, CD83, CD86, 4-1BBL co-stimulatory molecules, and CD70. K562 cells are commonly used as artificial antigen-presenting cells because they naturally lack HLA class I and II molecules, reducing unwanted allogeneic stimulation. Engineering them to overexpress co-stimulatory molecules such as CD80, CD83, CD86, and 4-1BBL provides the essential secondary signals required for robust NKT cell activation, proliferation, and survival. Adding CD70 further enhances activation through the CAR70–CD70 recognition, which promotes effector function and expansion of ^Allo^CAR70-NKT cells. Together, these modifications create a potent and standardized platform for driving strong and consistent ^Allo^CAR70-NKT cell stimulation *in vitro*.

51. Harvest the aAPCs, resuspend them in C10 medium at a density of 1–5 × 10^6^ cells/mL, and transfer the cells to a 50-mL conical tube. Maintain the tube on ice and expose the cells to 10,000 rads of irradiation.
52. Following irradiation, transfer the aAPCs to a biological safety cabinet.
53. Filter the aAPCs through a 70 μm sterile cell strainer to remove aggregates and count viable cells.
54. Centrifuge the aAPCs at 300 × g for 10 minutes, discard the supernatant, and combine them with the ^Allo^CAR70-NKT cells from the previous step at an ^Allo^CAR70-NKT:aAPC ratio of 1:1–1:2.
55. Resuspend the mixed cells in NKT cell expansion medium supplemented with 10 ng/mL human IL-7 and IL-15 to achieve a final density of 0.5–1 × 10^6^ cells/mL.

**CRITICAL:** IL-7 and IL-15 are critical cytokines for NKT cell survival, proliferation, and functional maturation. IL-7 supports early T/NKT-cell viability and homeostatic expansion, while IL-15 promotes robust proliferation, enhances cytotoxic function, and sustains long-term persistence of NKT cells.^4^ Supplementing the expansion medium with both cytokines creates an optimal environment that drives efficient ^Allo^CAR70-NKT cell growth while maintaining their functional phenotype during *in vitro* expansion.

56. Seed 2 mL of the ^Allo^CAR70-NKT/aAPC suspension per well into a non-precoated, non–tissue culture–treated Falcon 6-well plate, and culture for 2 weeks at 37°C, 5% CO_2_.
57. During the 2-week expansion period, passage the cells two to three times per week to maintain an optimal density of 0.5–1 × 10^6^ cells/mL.
58. At each passage, supplement the medium with 10 ng/mL IL-7 and 10 ng/mL IL-15 to sustain proliferation and viability of ^Allo^CAR70-NKT cells.
59. After 2 weeks, harvest and count the expanded ^Allo^CAR70-NKT cells.
60. Cryopreserve ^Allo^CAR70-NKT cells for downstream applications in CS10 freezing medium at a final concentration of 1 × 10^7^ cells/mL.

***Note:*** Stain cells with the following markers: NKT TCR 6B11, CD3, TCRαβ, CAR70, CD4, CD8, and viability dye (e506). The expected phenotype of differentiated ^Allo^CAR70-NKT cells is: >97% NKT cells (CD3^+^TCRαβ^+^6B11^+^CAR70^+^), cells primarily are CD4^-^CD8^-^ or CD4^-^CD8^+^, and >90% viability (e506^-^ population).

***Note:*** Besides flow cytometry, several other technologies can be used to monitor ^Allo^CAR70-NKT cell differentiation, including single cell RNA sequencing (scRNA-seq), fluorescence microscopy, bulk RNA sequencing, mass cytometry (CyTOF), and Assay for Transposase-Accessible Chromatin using sequencing (ATAC-seq).^13^

**Pause point:** Cryopreserve the cells in liquid nitrogen for subsequent functional testing; under these conditions, cells can be stably stored for up to 1 year.

### Evaluation of the *in vitro* antitumor efficacy of ^Allo^CAR70-NKT cells

**Timing: 2–3 days**

This section outlines the *in vitro* RCC tumor cell killing assay used to assess the cytotoxic activity and therapeutic potential of ^Allo^CAR70-NKT cells against RCC target cell lines.

61. Harvest FG-labeled RCC cells (e.g., 786-O-FG and ACHN-FG), resuspend them in C10 medium, and keep the suspension on ice until use.

**CRITICAL:** Ensure that the tumor cell population is fully GFP^+^, as reduced or lost GFP expression can result in inaccurate experimental outcomes.

62. Collect the ^Allo^CAR70-NKT cells, resuspend them in fresh C10 medium, and keep the cells on ice.

***Note:*** After thawing and resuspension, keep the cells chilled on ice or at 4°C to help preserve their viability. Sudden temperature changes or leaving the cells at elevated temperatures for too long can impair their integrity and function. Minimize any time spent at 20°C–25°C and transition to the next steps as quickly as possible to ensure maximal cell health.

***Note:*** For certain tumor cell lines that prefer alternative culture conditions, such as DMEM-based media, the co-culture assay should be performed using the medium that best supports tumor cell growth. In this setting, therapeutic cells, including ^Allo^CAR70-NKT cells, can be maintained in the tumor cell medium without issue for the duration of the assay.

63. In a Corning 96-well clear-bottom black plate, add tumor cells and ^Allo^CAR70-NKT effector cells at the indicated effector-to-target (E:T) ratios (0:1, 0.1:1, 0.5:1, 1:1, 2:1, 5:1, and 10:1). Troubleshooting 3.

***Note:*** Prepare four replicates for each E:T condition to ensure statistical reliability.

***Note:*** Because the CAR-T cell product is not 100% CAR-positive, all functional assays were normalized to the number of CAR^+^ cells rather than total cell count.

64. Incubate the co-culture plates at 37°C with 5% CO_2_ for 24–48 hours, depending on the experimental design.
65. After 24 hours of co-culture, add 100 μL of D-luciferin (150 μg/mL) to each well and incubate for 5 minutes at 20°C–25°C, protected from light.
66. Measure luciferase activity using an Infinite M1000 microplate reader (Tecan) to quantify residual tumor cell viability.

***Note:*** For the *in vitro* tumor cell killing assay, appropriate controls were included. We routinely used non–CAR-engineered PBMC-derived T cells as the negative control, and these cells did not exhibit effective cytotoxicity against RCC tumor cells.

## Expected outcomes

**Figure 1:**
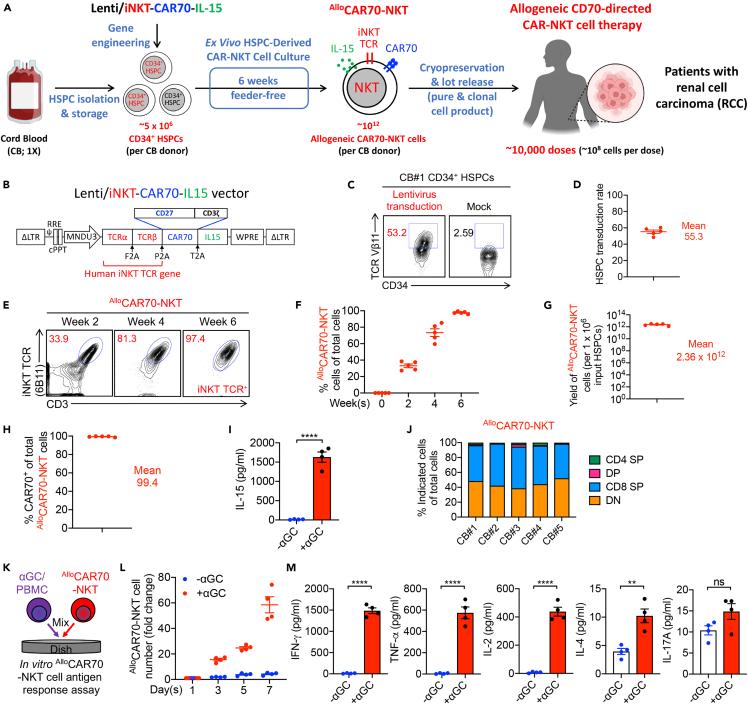
Generation of ^Allo^CAR70-NKT cells from HSPCs using a clinically guided culture method (A and B) Schematics showing the generation of ^Allo^CAR70-NKT cells (A) and the design of Lenti/iNKT-CAR70-IL-15 lentivector (B). (C) FACS detection of NKT TCR expression in lentivector-transduced CD34^+^ HSPCs. (D) Quantification of (C) (n = 5; n indicates different cord blood donors). (E) FACS monitoring of the generation of ^Allo^CAR70-NKT cells during the 6-week culture. (F) Percentage of ^Allo^CAR70-NKT cells in total live cells during the 6-week culture (n = 5; n indicates different cord blood donors). (G) Yield of ^Allo^CAR70-NKT cells (n = 5; n indicates different cord blood donors). (H) CAR70 expression on ^Allo^CAR70-NKT cells (n = 5; n indicates different cord blood donors). (I) ELISA measurements of IL-15 production by ^Allo^CAR70-NKT cells with or without αGC stimulation (n = 4). (J) CD4/CD8 subpopulations of ^Allo^CAR70-NKT cells. Data generated from 5 cord blood donors are shown. SP, single-positive; DP, double-positive; DN, double-negative. (K–M) Antigen responses of ^Allo^CAR70-NKT cells. ^Allo^CAR70-NKT cells were stimulated with/without αGC-loaded PBMCs for 1 week. (K) Experimental design. (L) Growth curve of ^Allo^CAR70-NKT cells (n = 4). (M) ELISA measurements of effector cytokine levels in the culture supernatants collected on day 5 (n = 4). Representative of over 5 experiments. Data are presented as the mean ± SEM. ns, not significant; ∗p < 0.05, ∗∗p < 0.01, ∗∗∗p < 0.001, and ∗∗∗∗p < 0.0001 by Student’s *t* test (I and M).

Human cord blood–derived CD34^+^ HSPCs were transduced with a lentiviral vector, Lenti/iNKT-CAR70-IL15, to generate CD70-targeting ^Allo^CAR70-NKT cells for RCC immunotherapy (Figures 1A and 1B). Following a 6-week *ex vivo* differentiation protocol, mature ^Allo^CAR70-NKT cells were successfully produced from all five cord blood donors tested. Each donor sample demonstrated a high and consistent lentivector transduction efficiency of approximately 50%, as determined by flow cytometry (Figures 1C and 1D). Because early-stage HSPCs lack surface CD3 expression, intracellular staining for TCR Vβ11 was employed to confirm successful NKT TCR expression (Figure 1C).

During *ex vivo* differentiation, progressive NKT lineage development was observed, with NKT cells comprising ∼30% of live cells by week 2, ∼80% by week 4, and >97% by week 6 (Figures 1E and 1F). Although the overall growth kinetics varied slightly among donors, all cultures exhibited similar developmental trajectories and phenotypic maturation. Importantly, the platform yielded exceptionally high numbers of ^Allo^CAR70-NKT cells: based on a linear calculation, approximately 1 × 10^12^ cells could be generated from a single cord blood unit containing 5 × 10^6^ HSPCs (Figure 1G). This level of scalability suggests that a single donor could produce sufficient cells to treat 1,000–10,000 patients, highlighting the high yield, purity, and allogeneic potential of this manufacturing process (Figure 1A).^15^

Flow cytometric profiling confirmed that nearly 100% of the differentiated cells expressed CAR70, owing to the use of a bicistronic lentivector encoding both the invariant NKT TCR and the CD70-specific CAR (Figure 1H). This vector design ensured that cells undergoing NKT-lineage differentiation co-expressed CAR70, leading to a homogeneous and phenotypically stable product. Following stimulation with α-galactosylceramide (αGC), a canonical NKT agonist, ^Allo^CAR70-NKT cells produced significant levels of human IL-15, confirming the stable genomic integration and functional expression of the IL-15 transgene (Figure 1I).

Subpopulation analysis revealed that ^Allo^CAR70-NKT cells consisted predominantly of CD4^-^CD8^-^ double-negative (DN) and CD8^+^ single-positive (SP) subsets—phenotypes commonly associated with robust cytotoxic activity and favorable antitumor efficacy (Figure 1J).^7^^,^^16^^,^^17^ Upon αGC stimulation *in vitro*, these cells exhibited vigorous proliferation and secreted Th1-associated cytokines, including IFN-γ, TNF-α, and IL-2, while producing minimal Th2 (IL-4) and Th17 (IL-17A) cytokines (Figures 1K–1M). This cytokine profile indicates a strong type 1 cytotoxic and proinflammatory polarization, suitable for tumor-targeted immune therapy.

**Figure 2:**
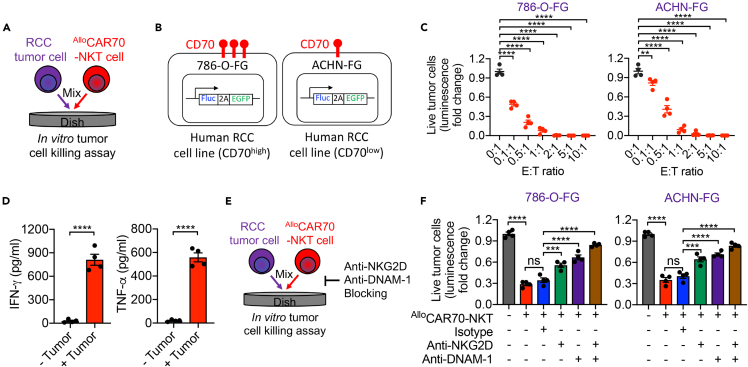
Evaluation of the *in vitro* antitumor efficacy of ^Allo^CAR70-NKT cells (A) Experimental design to study the *in vitro* antitumor efficacy of ^Allo^CAR70-NKT cells against human RCC cell lines. (B) Schematics showing the indicated human tumor cell lines. (C) Tumor cell killing data at 24 h (n = 4). (D) ELISA analyses of IFN-γ and TNF-α production by ^Allo^CAR70-NKT cells after 24-hour coculture with 786-O-FG cells (n = 4). (E) Experimental design to Study the tumor cell killing mechanisms of ^Allo^CAR70-NKT cells mediated by NKRs. (F) Tumor cell killing data at 24 h (E:T ratio = 0.5:1; n = 4). Representative of 3 experiments. Data are presented as the mean ± SEM. ns, not significant; ∗p < 0.05, ∗∗p < 0.01, ∗∗∗p < 0.001, and ∗∗∗∗p < 0.0001 by Student’s *t* test (D) or one-way ANOVA (C and F).

The cytotoxic potential of ^Allo^CAR70-NKT cells was evaluated using *in vitro* tumor cell killing assays against two RCC cell lines—786-O-FG (high CD70 expression) and ACHN-FG (low CD70 expression) (Figures 2A and 2B).^1^ After 24 hours of co-culture, ^Allo^CAR70-NKT cells mediated potent killing of both targets, accompanied by robust secretion of IFN-γ and TNF-α (Figures 2C and 2D). Notably, despite the low antigen density on ACHN-FG cells, substantial cytotoxicity was still observed, suggesting the involvement of additional innate-like recognition mechanisms. Blocking assays using anti-NKG2D and anti-DNAM-1 antibodies significantly reduced tumor lysis, indicating that natural killer receptors (NKRs) contribute synergistically with CAR70 signaling to mediate tumor elimination (Figures 2E and 2F).^18^ Consistently, ^Allo^CAR70-NKT cells also exhibited robust cytotoxicity against CD70-knockout RCC tumor cells, including 786-O-FG engineered by CRISPR/Cas9, further supporting that their antitumor activity is partially mediated through NKR-dependent mechanisms.^1^

Collectively, these findings demonstrate that ^Allo^CAR70-NKT cells possess high yield and purity, stable phenotype, strong Th1 cytokine polarization, and potent CAR-dependent and -independent antitumor activity, supporting their promise as a universal, off-the-shelf cell therapy platform for CD70^+^ malignancies such as RCC.

## Quantification and statistical analysis

All statistical analyses were conducted using GraphPad Prism 8. For comparisons between two groups, a two-tailed Student’s *t* test was applied. When comparing more than two groups, ordinary one-way ANOVA followed by Tukey’s post hoc test was used. Unless noted otherwise, data are displayed as mean ± SEM. In figures and legends, “n’’ indicates the number of independent biological replicates. Statistical significance was defined as p < 0.05. ns, not significant; ∗p < 0.05; ∗∗p < 0.01; ∗∗∗p < 0.001; ∗∗∗∗p < 0.0001.

## Limitations

This protocol describes the development of ^Allo^CAR70-NKT cells engineered to co-express the invariant NKT TCR, a CD70-targeting CAR, and IL-15 to enhance proliferation and persistence. While this design has demonstrated potent antitumor activity against human RCC in both *in vitro* and *in vivo* models,^1^^,^^19^ IL-15 overexpression may raise potential safety concerns, as suggested by observations from recent phase I clinical trials involving IL-15–augmented CAR-T cells in patients with hepatocellular carcinoma (HCC).^20^ Therefore, achieving an optimal balance between efficacy and safety is essential for future clinical translation.

To mitigate possible risks associated with uncontrolled cytokine activity or prolonged *in vivo* persistence, the incorporation of a suicide gene system, such as inducible caspase 9 (iCasp9) or herpes simplex virus thymidine kinase (HSV-TK), could be advantageous.^21^^,^^22^ These safety switches allow selective and rapid elimination of infused CAR-NKT cells upon administration of a small-molecule inducer (e.g., AP1903 for iCasp9), providing an additional layer of control over therapeutic activity. Implementing such a safety mechanism would enable dynamic regulation of ^Allo^CAR70-NKT cells, ensuring clinical safety without compromising therapeutic efficacy.

## Troubleshooting

### Problem 1

After thawing the CD34^+^ HSPC vial, the total number of cells is unexpectedly low, making it difficult to obtain a reliable viable cell count.

### Potential solution

When viable cell numbers are too low to count accurately, proceed by assessing overall cell integrity microscopically and estimate density based on visible cell concentration rather than relying on exact numeric values. If this issue recurs across multiple vials, evaluate the quality and consistency of the cryopreserved HSPC source, including shipping, storage stability, and freeze–thaw procedures. It may be necessary to verify the performance of different HSPC lots or adjust upstream cryopreservation practices to improve post-thaw viability. Recording viability trends across batches can help identify whether the issue originates from the donor material, cryostorage conditions, or handling workflow.

### Problem 2

The effective lentiviral MOI appears lower than expected, resulting in reduced transgene delivery to target cells.

### Potential solution

Verify that viral preparations retain functional potency and assess whether experimental variability—such as titer estimation, storage conditions, or cell health—may be contributing to reduced apparent MOI. Using these evaluations to adjust upstream quality control can help maintain consistent transduction performance.

### Problem 3

The killing assay may lack appropriate experimental controls, making it difficult to interpret whether observed tumor cell clearance is specifically attributable to ^Allo^CAR70-NKT cell activity.

### Potential solution

Include control effector populations that match the study design—such as non-engineered allogeneic NKT cells, healthy donor–derived T cells, or CAR70-engineered conventional T cells—to provide context for specificity and comparative function.^1^

## Resource availability

### Lead contact

For additional details or requests for materials, please contact the lead contact, Lili Yang (liliyang@ucla.edu), who will provide the necessary resources.

### Technical contact

For direct technical inquiries regarding this protocol, please contact the technical contact, Yan-Ruide Li (charlie.li@ucla.edu), who will answer all the questions.

### Materials availability

Human tumor cell lines generated in this study will be made available upon reasonable request.

### Data and code availability

This study did not generate new datasets or code.

## Acknowledgments

We thank the UCLA animal facility for providing animal support, the UCLA Translational Pathology Core Laboratory (TPCL) for providing histology support, and the UCLA CFAR Virology Core for providing human cells. This work was supported by a Partnering Opportunity for Discovery Stage Research Projects award and Partnering Opportunity for Translational Research Projects awards from the California Institute for Regenerative Medicine (DISC2-11157, DISC2-13015, TRAN1-12250, and TRAN1-16050 to L.Y.), a Department of Defense Kidney Cancer Research Program award (KC230215 to L.Y.), and a UCLA BSCRC Innovation Grant. L.Y. is a member of UCLA Parker Institute for Cancer Immunotherapy (PICI). Y.-R.L. is a postdoctoral fellow supported by a UCLA Chancellor’s Award for Postdoctoral Research and a UCLA Goodman-Luskin Microbiome Center Collaborative Research Fellowship award. Some figures were created with BioRender (biorender.com).

## Author contributions

Y.-R.L. and L.Y. designed the experiments, analyzed the data, and wrote the manuscript.

## Declaration of interests

Y.-R.L. and L.Y. are inventors on patents related to this manuscript. L.Y. is a scientific advisor to Alzchem and Amberstone Biosciences and a co-founder, stockholder, and advisory board member of Appia Bio. None of the declared companies contributed to or directed any of the writing of this manuscript.

## References

[1] Li Y.-R., Hu J., Li Z., Zhu E., Chen Y., Halladay T., Shen X., Fang Y., Zhu Y., Lyu Z. (2025). Multimodal targeting of metastatic renal cell carcinoma via CD70-directed allogeneic CAR-NKT cells. Cell Rep. Med..

[2] Wei W., Grünwald V., Herrmann K. (2025). CD70-targeted cancer theranostics: Progress and challenges. Med.

[3] Grewal I.S. (2008). CD70 as a therapeutic target in human malignancies. Expert Opin. Ther. Targets.

[4] Zhou Y., Li Y.-R., Zeng S., Yang L. (2021). Methods for Studying Mouse and Human Invariant Natural Killer T Cells. Methods Mol. Biol..

[5] Chen Y., Zhu Y., Kramer A., Fang Y., Wilson M., Li Y.-R., Yang L. (2023). Genetic engineering strategies to enhance antitumor reactivity and reduce alloreactivity for allogeneic cell-based cancer therapy. Front. Med..

[6] Chen Y., Niu S., Li Y.-R., Yang L. (2025). Innovative gene engineering strategies to address tumor antigen escape in cell therapy. J. Transl. Med..

[7] Courtney A.N., Tian G., Metelitsa L.S. (2023). Natural killer T cells and other innate-like T lymphocytes as emerging platforms for allogeneic cancer cell therapy. Blood.

[8] Cortés-Selva D., Dasgupta B., Singh S., Grewal I.S. (2021). Innate and Innate-Like Cells: The Future of Chimeric Antigen Receptor (CAR) Cell Therapy. Trends Pharmacol. Sci..

[9] Heczey A., Liu D., Tian G., Courtney A.N., Wei J., Marinova E., Gao X., Guo L., Yvon E., Hicks J. (2014). Invariant NKT cells with chimeric antigen receptor provide a novel platform for safe and effective cancer immunotherapy. Blood.

[10] Li Y.-R., Zhou Y., Kim Y.J., Zhu Y., Ma F., Yu J., Wang Y.-C., Chen X., Li Z., Zeng S. (2021). Development of allogeneic HSC-engineered iNKT cells for off-the-shelf cancer immunotherapy. Cell Rep. Med..

[11] Giannoni F., Hardee C.L., Wherley J., Gschweng E., Senadheera S., Kaufman M.L., Chan R., Bahner I., Gersuk V., Wang X. (2013). Allelic exclusion and peripheral reconstitution by TCR transgenic T cells arising from transduced human hematopoietic stem/progenitor cells. Mol. Ther..

[12] Zhu Y., Smith D.J., Zhou Y., Li Y.R., Yu J., Lee D., Wang Y.C., Di Biase S., Wang X., Hardoy C. (2019). Development of Hematopoietic Stem Cell-Engineered Invariant Natural Killer T Cell Therapy for Cancer. Cell Stem Cell.

[13] Li Y.-R., Zhou Y., Yu J., Kim Y.J., Li M., Lee D., Zhou K., Chen Y., Zhu Y., Wang Y.-C. (2025). Generation of allogeneic CAR-NKT cells from hematopoietic stem and progenitor cells using a clinically guided culture method. Nat. Biotechnol..

[14] Li Y.-R., Zhou K., Lee D., Zhu Y., Halladay T., Yu J., Zhou Y., Lyu Z., Fang Y., Chen Y. (2025). Generating allogeneic CAR-NKT cells for off-the-shelf cancer immunotherapy with genetically engineered HSP cells and feeder-free differentiation culture. Nat. Protoc..

[15] Li Y.-R., Zhu Y., Fang Y., Lyu Z., Yang L. (2025). Emerging trends in clinical allogeneic CAR cell therapy. Med.

[16] Lee P.T., Benlagha K., Teyton L., Bendelac A. (2002). Distinct functional lineages of human Vα24 natural killer T cells. J. Exp. Med..

[17] Liu J., Hill B.J., Darko S., Song K., Quigley M.F., Asher T.E., Morita Y., Greenaway H.Y., Venturi V., Douek D.C. (2019). The peripheral differentiation of human natural killer T cells. Immunol. Cell Biol..

[18] Li Y.-R., Li Z., Zhu Y., Li M., Chen Y., Lee D., Ochoa C.J., Singh T., DiBernardo G., Guo W. (2025). Overcoming ovarian cancer resistance and evasion to CAR-T cell therapy by harnessing allogeneic CAR-NKT cells. Med.

[19] Li Y.r., Hu J., Yang L., Wu L., Chin A.I. (2025). 54Allogeneic HSPC-engineered CD70-directed CAR-NKT cells for renal cell carcinoma targeting tumor, microenvironment, and alloreactive T cells. Oncologist.

[20] Steffin D., Ghatwai N., Montalbano A., Rathi P., Courtney A.N., Arnett A.B., Fleurence J., Sweidan R., Wang T., Zhang H. (2025). Interleukin-15-armoured GPC3 CAR T cells for patients with solid cancers. Nature.

[21] Li Y.-R., Dunn Z.S., Yu Y., Li M., Wang P., Yang L. (2023). Advancing cell-based cancer immunotherapy through stem cell engineering. Cell Stem Cell.

[22] Li Y.-R., Zhou Y., Yu J., Zhu Y., Lee D., Zhu E., Li Z., Kim Y.J., Zhou K., Fang Y. (2024). Engineering Allorejection-Resistant CAR-NKT Cells from Hematopoietic Stem Cells for Off-The-Shelf Cancer Immunotherapy. Mol. Ther..

